# Impact of intraoperative fluid administration on outcome in patients undergoing robotic-assisted laparoscopic prostatectomy – a retrospective analysis

**DOI:** 10.1186/1471-2253-14-61

**Published:** 2014-07-30

**Authors:** Tobias Piegeler, Pamela Dreessen, Sereina M Graber, Sarah R Haile, Daniel Max Schmid, Beatrice Beck-Schimmer

**Affiliations:** 1 Institute of Anesthesiology, University Hospital Zurich, Raemistrasse 100, 8091 Zurich, Switzerland; 2 Surgical Intensive Care Medicine, University Hospital Zurich, Raemistrasse 100, 8091 Zurich, Switzerland; 3 Institute of Physiology and Center for Integrative Human Physiology, University of Zurich, Winterthurerstrasse 190, 8057 Zurich, Switzerland; 4 Institute for Social and Preventive Medicine, Division of Biostatistics, University of Zurich, Hirschengraben 84, 8001 Zurich, Switzerland; 5 Department of Urology, University Hospital Zurich, Frauenklinikstrasse 10, 8091 Zurich, Switzerland

**Keywords:** Hospitalization, Perioperative care, Colloids, Crystalloid solutions

## Abstract

**Background:**

Robotic-assisted laparoscopic prostatectomy (RALP) gained much popularity during the last decade. Although the influence of intraoperative fluid management on patients’ outcome has been largely discussed in general, its impact on perioperative complications and length of hospitalization in patients undergoing RALP has not been examined so far. We hypothesized that a more restrictive fluid management might lead to a shortened length of hospitalization and a decreased rate of complications in our patients.

**Methods:**

Retrospective analysis of data of 182 patients undergoing RALP at an University Hospital (first series of RALP performed at the center).

**Results:**

The amount of fluid administered was initially normalized for body mass index of the patient and the duration of the operation and additionally corrected for age and the interaction of these variables. The application of crystalloids (multiple linear regression model, estimate = -0.044, p = 0.734) had no effect on the length of hospitalization, whereas a negative effect was found for colloids (estimate = -8.317, p = 0.021). Additionally, a significant interaction term between age and the amount of colloid applied (estimate = 0.129, p = 0.028) was calculated. Evaluation of the influence of intraoperative fluid administration using multiple logistic regression models corrected for body mass index, duration of the surgery and additionally for age revealed a negative effect of crystalloids on the incidence of an anastomotic leak between bladder and urethra (estimate = -23.860, p = 0.017), with a significant interaction term between age and the amount of crystalloids (estimate = 0.396, p = 0.0134). Colloids had no significant effect on this particular complication (estimate = 1.887, p = 0.524). Intraoperative blood loss did not alter the incidence of an anastomotic leak (estimate = 0.001, p = 0.086), nor did it affect the length of hospitalization (estimate = 0.0001, p = 0.351).

**Conclusions:**

In accordance to the findings of our study, we suggest that a standardized, more restrictive fluid management might be beneficial in patients undergoing RALP. In older patients this measure would be able to shorten the length of hospitalization and to decrease the incidence of anastomosis leakage as a major complication.

## Background

Since its introduction in 2001 [1], robotic-assisted laparoscopic prostatectomy (RALP) has gained much popularity and is increasingly used as a minimally-invasive surgical treatment for patients with localized prostate cancer in many centers throughout the world [2]. Several previous studies have shown that the technique is feasible and leads to fewer complications than the traditional approach, known as open retropubic radical prostatectomy [3-5]. Due to the fact that patients have to be placed in steep Trendelenburg position during RALP, maintaining homeostasis might be challenging for the anesthesiologist [6]. The influence of Trendelenburg position on cardio-/cerebrovascular and respiratory homeostasis has been studied recently [7]. However, to our knowledge the impact of intraoperative fluid administration on outcome in RALP has not been addressed.

Perioperative fluid management and its influence on postoperative complications and patients’ outcome in general is a crucial aspect in our daily clinical practice as anesthesiologists. There is evidence that a more restrictive, protocol-based fluid management – best with goal-directed fluid administration – might lead to fewer complications, e.g. after abdominal surgery or elective colorectal resection, respectively [8-10]. It has also been reported that intraoperative administration of colloids reduced postoperative nausea and vomiting (PONV) and improved patients’ outcome after major non-cardiac surgery compared to crystalloids [11]. However, the definition of liberal vs. restrictive fluid regimens is still inconsistent: The authors of a recent review examined seven randomized trials comparing liberal vs. restrictive fluid management in patients undergoing major surgery and reported very large margins of administered fluids in both groups [12]. Based on the finding that a liberal regimen in one of the examined trials was reported to be only 10 ml higher [13] than a restrictive one in another trial [8], Doherty and Buggy concluded that due to the lack of well-defined liberal or restrictive protocols, respectively, the two expressions (liberal and restrictive) might be used and understood differently in different institutions [14].

In our study, we retrospectively analyzed data of 182 patients to assess whether intraoperative fluid management – administration of crystalloids or colloids – had an influence on the incidence of postoperative complications and the length of hospitalization. These patients were among the first ones undergoing RALP in our University Hospital. Patients who received one or more erythrocyte concentrates were excluded from the analysis (n = 12).

We hypothesized that a less aggressive intraoperative fluid management might lead to a shortening of the length of hospitalization as well as an amelioration of the patients’ clinical outcome.

## Methods

After approval by the local ethics committee (Kantonale Ethikkommission, Stampfenbachstrasse 121, 8090 Zurich, Switzerland; study protocol KEK-ZH number: 2010-0043/0) perioperative data of 182 male patients (ASA classification I – III) with localized prostate cancer undergoing elective RALP between 2005 and 2008 at University Hospital Zurich, Switzerland, were analyzed retrospectively. This patient collective represents the first patients undergoing this particular prostatectomy technique at this center with four different surgeons performing this surgery. The following patients were considered not to be eligible for the study: patients who received erythrocyte concentrates in the perioperative period or patients with a history of chronic obstructive pulmonary disease stage GOLD IV, congestive heart failure stage NYHA IV or ASA classification IV.

Patients were given different types of fluids by the anesthesiologist intraoperatively: crystalloid (Ringerfundin® B. Braun, B. Braun Medical AG, Switzerland) or colloid solutions, either hydroxyethylstarch (HES) or modified fluid gelatin (Physiogel® 4%, B. Braun Medical AG, Switzerland). From 2005 to 2006, HES has been administered as Voluven® 6% (HES 130/0.4, Fresenius Kabi AG, Switzerland) and from 2006 to 2008 as Tetraspan® 6% (HES 130/0.42, B. Braun Medical AG, Switzerland). There has been no protocol to be followed by the anesthesiologist concerning the administration of crystalloids or colloids. Fluid administration was left to the discretion of the anesthesiologists who assessed the fluid status of the patients mainly based on blood pressure in combination with heart rate, as urine output could not be measured due to the nature of the surgical procedure. Also, assessment of central venous pressure through central venous lines is not part of the anesthesia routine in our institution in patients undergoing RALP.

Data was collected from the anesthesia protocol as well as from a database provided by the Department of Urology at the University Hospital Zurich and included patient characteristics, intraoperative fluid administration and perioperative complications.

As primary endpoint we defined the length of hospitalization in days. Secondary endpoint was the clinical outcome, i.e. the rate of (specific) perioperative complications.

### Statistical analysis

Summary statistics are given for all variables. The effect of different types of fluids on the length of hospitalization was examined using parametric multiple linear regression models. To avoid potential confounding variables we calculated the amount of fluid administered per unit of the individual patient’s body mass index (BMI) per minute (of duration of the surgery; ml fluid/unit BMI*minute operation duration). The length of hospitalization (days) was log_e_ transformed. Additionally, our analysis was corrected for the age of the patients.

To test the effects of the amount of crystalloids and colloids on specific complications, multiple logistic regression models were used. Again, the amount of colloids and crystalloids have the unit ml per unit BMI and minute operation duration (ml fluid/unit BMI*minute operation duration) in order to correct the amounts of applied fluids for the individual patient’s body size and the duration of the surgery. Additionally the analyses were corrected for age. Interaction terms are included in the model if significant. To evaluate the different models for the best fit, we used Akaike’s information criterion (AIC). In our Results section we only report the models with the best fit as well as with a significant influence of the fluid applied. All other models are not shown. A comparison of estimated blood loss between two groups of patients of different ages (70–80 years old versus less than 70 years of age) was conducted using a Wilcoxon rank sum test with continuity correction. A *p*-value of <0.05 was considered to be statistically significant. All analysis was performed in the R programming language [15].

## Results

### Patient and perioperative characteristics

Perioperative characteristics of 182 male patients with prostate cancer who underwent RALP were examined (Table 1). At the time of surgery, patients were 64 years old (median, range 44 – 78), had a body mass index (BMI) of 26.4 (19.0 – 37.8) and a median prostate weight of 42 g (8 – 197). During a median duration of surgery of 240 minutes (135 – 515) patients (n = 181) received a median total amount of 3600 ml fluids (1200 – 9000) intraoperatively, with a median amount of 3000 ml (1000 – 8000) crystalloids. Colloids were given to 143 patients and corresponding to the whole study group, a median of 500 ml (0 – 2000) has been administered. The median blood loss during surgery was 400 ml (100 – 2000). The preoperative median concentration of hemoglobin was 14.7 g/dl (12.0 – 17.2). Postoperative measures revealed a median hemoglobin concentration of 11.5 g/dl (6.6 – 14.8). The median length of hospitalization was 8 days (4 – 23).

**Table 1:** Patient characteristics and continuous variables

***Variable***	***n***	**#** ***NA***	***Min***	***Max***	***Median***	***Trimmed mean***	***s***	***q***_***1***_	***q***_***3***_	***IQR***
Age [years]	182	0	44	78	64	63	6	59	67	8
BMI	143	39	19.0	37.8	26.4	26.4	3.3	24.4	28.1	3.7
Blood loss [ml]	181	1	100	2000	400	426.8	315.2	300	600	300
Prostate weight [g]	182	0	8	197	42	45.4	24.5	34	55.5	22
Hospital stay [days]	182	0	4	23	8	8.1	3.1	7	9	2
Total volume of fluids [ml]	181	1	1200	9000	3600	3677	1325	2900	4400	1500
Duration of operation [min]	182	0	135	515	240	254.3	72.4	210	300	90
Total volume of colloids [ml]	181	1	0	2000	500	595.1	443.8	500	1000	500
Total volume of crystalloids [ml]	181	1	1000	8000	3000	3055.8	1183.4	2300	3600	1300
Hemoglobin concentration preoperative [mg/dl]	182	0	12.0	17.2	14.7	14.7	1.0	14.1	15.3	1.2
Hemoglobin concentration postoperative [mg/dl]	178	4	6.6	14.8	11.5	11.3	1.4	10.5	12.1	1.6

Patient characteristics and continuous variables; number of patients (n), number of missing values (#NA), minimum (Min), maximum (Max), standard deviation (s), 1st quartile (q1), 3rd quartile (q3), and interquartile range (IQR); BMI = body mass index.

### Perioperative events and complications

Several events and complications have been recorded during the perioperative period (Table 2). Two patients (1.1%) suffered from a postoperative cardiac event: one patient developed a cardiac pulmonary edema and atrial fibrillation due to pre-existing coronary artery disease. A second patient had symptoms of angina pectoris with negative T spikes in leads V_3_-V_5_ (a coronary catheterisation revealed generally coronary sclerosis with left ventricular hypertrophy). An exacerbation of the pre-existing arterial hypertension was observed in a third patient. One patient (0.55%) developed deep venous thrombosis in the postoperative phase. Postoperative nausea and vomiting had a low incidence (2 patients, 1.1%).

**Table 2:** Rates of perioperative complications

**Complications**	**n (%)**
**Organ specific complications**	
Cardiac	2 (1.1)
Vascular	1 (0.55)
Gastrointestinal	
PONV	2 (1.1)
**Urologic**/**surgical complications**	
Anastomotic leak (bladder – urethra)	30 (16.5)
Leakage (urethra)	6 (3.3)
Tamponade (bladder)	2 (1.1)
Combined injury (leakage anastomosis and tamponade bladder)	1 (0.55)
Epididymitis	2 (1.1)
Limited micturition	1 (0.55)
Constriction (urethra)	1 (0.55)
Constriction (ureter)	1 (0.55)
Wound infection	1 (0.55)
**Re**-**operation**	3 (1.6)
**Death**	0 (0.0)

PONV = postoperative nausea and vomiting.

The most common urological complication was an anastomotic leak between the bladder and the urethra, which was diagnosed via cystoscopy and occurred in 30 patients (16.5% of total study group, Table 2). Postoperative leakage of the urethra was found in 6 patients (3.3%), a tamponade of the bladder was observed in 2 patients (1.1%) and epididymitis in 2 patients (0.55%). Only 1 patient each (0.55%) had a constriction of the urethra or the ureter, limited micturition or a leakage of the anastomosis combined with bladder tamponade. Wound infection had a low incidence (1 patient, 0.55%). A total of 3 patients (1.6%) had to undergo a second operation because of surgical complications. None of the 182 patients died during the perioperative period.

### Influence of intraoperative fluids on the length of hospitalization

To investigate whether intraoperative fluid administration influences the length of hospitalization after RALP, we analyzed the amount of the two different fluids (crystalloids and colloids) using parametric multiple linear regression models additionally corrected for age. The amount of fluid administered was calculated as ml fluid per unit of the BMI and minute of the duration of the surgery (ml fluid/unit BMI*minute operation duration) in order to eliminate potentially confounding variables. The length of hospitalization was log_e_ transformed.

**Figure 1:**
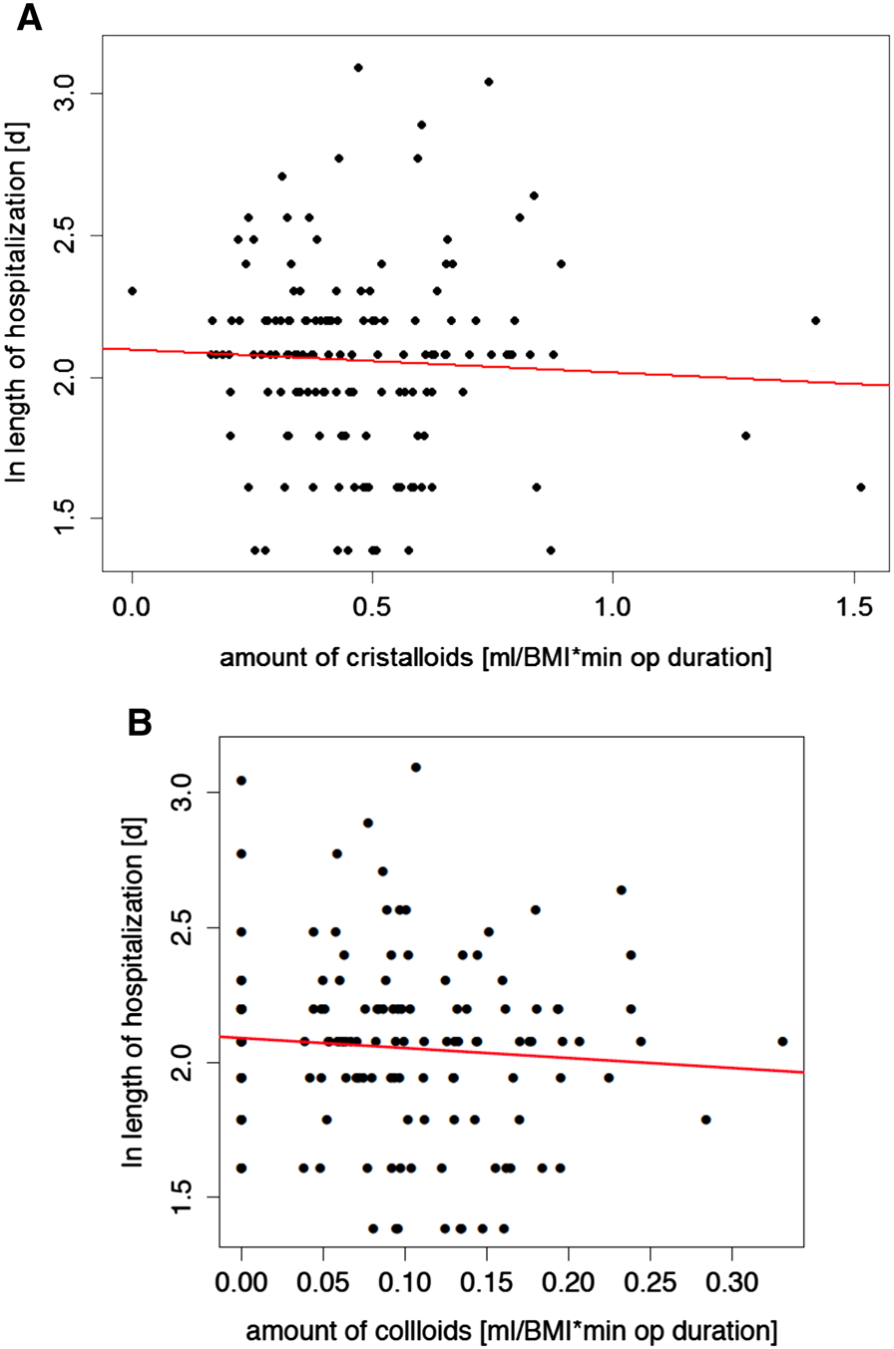
**Influence of intraoperative fluid administration on the length of hospitalization after robotic**-**assisted laparoscopic prostatectomy.** The amount of fluids administered (crystalloids **(A.)** and colloids **(B.)**) was corrected for body mass index (BMI) and the duration of surgery (ml fluid/unit BMI*minute of operation duration), the length of hospitalization in days (d) was log_e_ transformed. Analysis was conducted using parametric multiple linear regression models additionally corrected for age.

The amount of crystalloid applied had no influence on the length of hospitalization (multiple linear regression model, estimate = -0.044, p = 0.734, Figure 1A and Table 3). In turn, the analysis of the effect of the amount of colloids applied on the length hospitalization revealed a significant negative impact (estimate = -8.317, p = 0.021, Figure 1B and Table 3). Additionally, the interaction term between the amount of colloids and the age of the patient is significant (estimate = 0.129, p = 0.028, Table 3). Taken together, these findings indicate that younger patients (around 45 years old) show a *negative* relationship between the duration of the hospitalization and the amount of colloids, meaning they stayed in the hospital for a shorter period of time, if they received more colloids intraoperatively. However, for older patients (70 – 80 years old, n = 20 in our study), there is a *positive* relationship between the two parameters colloid application and duration of hospitalization: the more colloids these patients were infused, the longer they were hospitalized (estimate = 2.256, p = 0.034, Table 3).

**Table 3:** **Influence of intraoperative fluid administration on the length of hospitalization after robotic**-**assisted laparoscopic prostatectomy**

***Type of fluid***	***AIC***	***n***	***ml fluid*/ *unit BMI *** * ***minute operation duration***			***Age***			*ml fluid*/unit BMI*minute operation duration : Age		
			***Estimate***	***Std*. *Error***	***p*-*value***	***Estimate***	***Std*. *Error***	***p*-*value***	***Estimate***	***Std*. *Error***	***p*-*value***
**CRYSTALLOID**	96.08	181	-0.044	0.129	0.734	0.005	0.004	0.226			
**COLLOID**	91.93	143	-8.317	3.571	0.021*	-0.005	0.007	0.420	0.129	0.058	0.028*
**COLLOID**	17.93	20	2.656	1.163	0.034*						
Patients: Age 70–80 y											

Influence of intraoperative fluid administration (corrected for BMI and the duration of surgery; ml fluid/unit BMI*minute of operation duration) on the length of hospitalization after robotic-assisted laparoscopic prostatectomy. Analysis was conducted using parametric multiple linear regression models additionally corrected for age and the interaction term between the amount of fluids administered and the age of the patient. Significant effects (p < 0.05) are marked with “*” and presented in bold font. BMI = body mass index; Std. Error = standard error; y = years; AIC = Akaike’s information criterion.

### Influence of intraoperative fluids on the incidence of specific complications

Mulitple logistic regression models were used to examine the effect of the application of crystalloids and colloids on the incidence of specific complications, which occurred during the perioperative period and that are listed in Table 2. During the analysis we again calculated the amount of fluid in relation to the patients BMI and the duration of the operation (ml fluid/unit BMI*minute operation duration) and additionally corrected for the patient’s age. In Table 4 we report the results of these analyses: The intraoperative application of colloids did not influence the incidence of an insufficient anastomosis between urethra and bladder (multiple linear regression model, estimate = 1.887, p = 0.524). However, there was a significant negative effect (estimate = -23.860, p = 0.0167) of the amount of crystalloids on complications with the anastomosis. We again also found a significant interaction term (estimate = 0.396, p = 0.0134) between the amount of crystalloids and the age of the patient, meaning that for young patients (around 45 years old) there is a *negative* effect of the amount of crystalloids on complications with the anastomosis: the *more* crystalloids these patients received, the *lower* the probability of having a leaking anastomosis. In turn, rather old patients (70 – 80 years old) did not benefit from crystalloid application and showed a *positive* effect: *increasing* amounts of crystalloids also *increased* the incidence of a complication with the anastomosis. The incidence of all other specific complications was not altered significantly by the application of neither crystalloids nor colloids.

**Table 4:** **Influence of intraoperative fluid administration on specific complications after robotic**-**assisted laparoscopic prostatectomy**

***Type of fluid***	***AIC***	***Variable***/***Complication***	***ml fluid*/ *unit BMI *** * ***minute operation duration***			***Age***			***ml fluid*/ *unit BMI *** * ***minute operation duration : Age***		
			*Estimate*	*Std. Error*	*p*-*value*	*Estimate*	*Std. Error*	*p*-*value*	*Estimate*	*Std. Error*	*p*-*value*
**CRYSTALLOID**	138.42	Anastomosis	-23.860	9.9716	0.0167*	-0.2398	0.0916	0.0088*	0.3955	0.1599	0.0134*
**COLLOID**	143.24	Anastomosis	1.887	2.9640	0.524	-0.033	0.032	0.302			

Influence of intraoperative fluid administration (corrected for BMI and the duration of surgery; ml fluid/unit BMI*minute of operation duration) on specific complications in the perioperative period after robotic-assisted laparoscopic prostatectomy. Analysis was conducted using parametric multiple logistic regression models additionally corrected for age and the interaction term between the amount of fluids administered and the age of the patient. Significant effects (p < 0.05) are marked with “*” and presented in bold font. *BMI* = body mass index; *Std. Error* = standard error; *AIC* = Akaike’s information criterion.

### Influence of intraoperative blood loss on the length of hospitalization and the incidence of specific complications

Patients who received a perioperative blood transfusion were not included in our analysis. However, to exclude the possibility that the intraoperative blood loss of the remaining patients’ might affect their outcome, we evaluated the impact of the blood loss on both the length of hospitalization as well as on the incidence specific complications. Apparently, the length of hospitalization was not influenced by the intraoperative blood loss (estimate = 0.0001, p = 0.351, Table 5), even if additionally corrected for the age of the patient (estimate = 0.007, p = 0.119, Table 5). We also did not find a correlation between the incidence of an anastomotic leak and intraoperative blood loss (estimate = 0.001, p = 0.086, Table 5). Also, there was no significant difference in estimated blood loss between the group of patients 70 – 80 years old and the patients less than 70 years of age (Wilcoxon rank sum test with continuity correction, W = 1900, p = 0.948).

**Table 5:** **Influence of intraoperative blood loss on the length of hospitalization and the incidence of a leaking anastomosis as a major specific complication after robotic**-**assisted laparoscopic prostatectomy**

***Variable***	***AIC***	***Variable*/*Complication***	***ml blood loss***			***Age***		
			***Estimate***	***Std*. *Error***	***p*-*value***	***Estimate***	***Std*. *Error***	***p*-*value***
**BLOOD LOSS**	94.69	Length of hospitalization	0.0001	0.0001	0.351	0.0066	0.0042	0.119
**BLOOD LOSS**	141.96	Anastomosis	0.001	0.0323	0.0862	-0.0262	0.0323	0.418

Influence of intraoperative blood loss (ml) on the length of hospitalization and the incidence of a leaking anastomosis as a major specific complication in the perioperative period after robotic-assisted laparoscopic prostatectomy. Analysis was conducted using parametric multiple logistic regression models additionally corrected for age and the interaction term between the amount of fluids administered and the age of the patient. *Std. Error* = standard error; *AIC* = Akaike’s information criterion.

## Discussion

Major findings of the current retrospective study are that administration of increasing amount of colloids to older patients (70 – 80 years old) was associated with an increase in the length of hospitalization after RALP. The statistical analysis of this particular correlation revealed an estimate of 2.256 (standard error 1.163), suggesting that patients at the age of 70–80 years will be hospitalized about 2 days longer per ml colloid/unit BMI and minute duration of the surgery they received. However, younger patients (around 45 years old) seemed to benefit from colloid administration during surgery, as the length of hospitalization was reduced. Crystalloids did not have an influence on the length of hospitalization in patients undergoing RALP. In addition, we were able to demonstrate a statistically significant impact of the intraoperative administration of crystalloids on complications regarding the anastomosis between urethra and bladder: in older patients, administration of increasing amounts of crystalloids was also associated with an increase in the incidence of anastomotic leaks, whereas younger patients showed a reduction in anastomotic leaks after crystalloid administration.

These results are comparable to the findings of a recent study, showing that the use of a restrictive fluid management reduces complications after colorectal surgery compared to a standard fluid regimen [8]. In this particular study the restricted regimen focused on maintaining pre-operative body weight, while the standard regimen represented everyday practice [8]. Postoperative complications were defined as primary outcome. Intraoperative fluid application in the restricted group was 2740 ml (median), while 5388 ml were applied to the patients in the liberal group, resulting in a significant reduction of postoperative complications in the restricted group. A dose-dependent complication rate was observed as well: The number of cardiopulmonary complications decreased from 24% to 7% and tissue-healing complications from 31% to 16%. Anastomosis leakage was observed in 1 patient in the restricted versus 4 in the liberal group.

Due to their composition, crystalloids distribute within the whole extracellular space [16]. One fifth of the applied volume stays in the vascular compartment, four fifths shift to the interstitial space [16]. Liberal crystalloid administration may therefore lead to interstitial edema formation, which is known to impair intestinal anastomotic stability in rats [17], whereas colloids compared to crystalloids seem to be advantageous concerning intestinal anastomotic healing after high-volume fluid administration in the same animals [18]. The results of our study confirm these findings, as crystalloids were associated with a higher incidence in anastomotic leakage in older patients, whereas colloids had no significant effect.

Recent studies suggest an individualization of fluid therapy (goal-directed fluid management) rather than restriction. In a prospective randomized study in patients undergoing high-risk surgery, stroke volume was maximized in the intervention group by minimizing ventilator-induced variation in arterial pulse pressure compared to an untreated group [19]. Patients in the intervention group received more fluids (4618 +/- 1557 versus 1,694 +/- 705 ml (mean +/- SD)) but the complication rate as well as the number of postoperative complications was lowered [19]. Another goal-directed approach of fluid management was tested in pigs undergoing colon anastomosis [9]. The authors compared microcirculation and tissue oxygen tension between three different fluid regimens: one group with a restricted protocol, just receiving a continuous infusion of lactated Ringer’s solution (RL, 3 ml/kg/h) and two groups with the same continuous infusion rate with lactated Ringer’s solution, but with a 250 ml bolus administration of either crystalloids or colloids, if mixed venous oxygen saturation dropped below 60%. After 4 hours of treatment, the tissue oxygen tension in peri-anastomotic colon was significantly higher in both goal-directed groups, whereas the group with colloid boluses showed even significantly higher values than the group with crystalloid boluses (116% control vs. 147% crystalloid bolus group vs. 245% colloid bolus group) [9]. Goal-directed fluid therapy with colloids might therefore be beneficial concerning microcirculation after colon resection and anastomosis.

Another randomized trial has shown an improvement in postoperative pulmonary function (forced vital capacity, forced expiratory volume in first second) and exercise capacity after laparoscopic cholecystectomy by a more liberal crystalloid regimen (40 ml/kg lactated Ringer’s solution versus 15 ml/kg) and no use of colloids [20]. A contrary effect regarding postoperative pulmonary function could be revealed after fast-track colonic surgery [21]. A more restrictive fluid regimen (median 1640 ml) seemed to be beneficial in immediate postoperative pulmonary function compared to liberal fluid administration (median 5050 ml). However, overall physiological recovery was comparable in both groups, and postoperative morbidity tended to be worse in the restrictive group (6 patients with complications in the restrictive versus 1 in the liberal group) [21]. An important confounder of these studies might be the difference in surgery, the intraoperative position of the patient, the surgical technique (laparoscopic or open surgery), etc. Due to the retrospective character of our study, immediate postoperative pulmonary function was not assessed as this is not a routine procedure. Additionally, no pulmonary complications were registered in the postoperative phase of all patients.

The amount of fluids administered to the patients in our study (median of 3600 ml total, median of 3000 ml crystalloids) was relatively high compared to previous publications. A recent study reported a series of 575 patients undergoing RALP, who only received a median of 1600 ml of crystalloids [22]. In a review of 1500 cases the authors stated that pre- and intraoperative intravenous fluid administration should be kept below 2000 ml to minimize a possible impairment of the operative field by excessive urine output as well as facial edema due to the Trendelenburg position [23]. Additionally, the authors recommended a restoration of the patients fluid depletion throughout the procedure with a rapidly infused bolus of 1 liter lactated Ringer’s solution after the patient has been put back into the supine position followed by a continuous infusion of 150 ml per hour for the next 12–24 hours according to the patient’s volume status [23]. In our study, the administration of fluids was left to the discretion of the anesthesiologist. These cases were the first to be conducted at our institution. The relatively high median amount of fluids administered might therefore be explained by the learning curve of the different anesthesiologists who had to adjust and transfer the regimen of fluid resuscitation from an open to a laparoscopic prostatectomy procedure.

Additionally, the surgeon’s experience and the according learning curve also determine the rate of postoperative complications [24,25]. The incidence of an anastomotic leak was 16.5% (30 of 182 patients) in our study. This value seems to be rather high compared to an incidence of 1.4% for this particular complication in another study of 2500 RALPs conducted by a single surgeon. We report a median estimated blood loss of 400 ml. Other large studies, however, indicated a median blood loss ranging from 50 to 200 ml for RALP [3,22,26]. A median blood loss of 376 ml, comparable to the value found in our study, was reported in an analysis of the first laparoscopic radical prostatectomies at a single institution [27]. In our study, four different surgeons at different levels of experience were involved. The number of cases a surgeon has to perform in order to significantly decrease the levels of complications has been postulated as 150 in another single-surgeon study of 200 cases [25]. As already mentioned the analyzed patients were the first ones undergoing RALP at our institution. Taken together with data from the literature, the relatively high incidence of particular complications and high amount of estimated blood loss might possibly be explained by the learning curve of the surgeons starting to conduct the procedure. Additionally, analysis of the impact of intraoperative blood loss on the length of hospitalization and on the incidence of specific complications did not reveal any significant effects in our patients.

Possible limitations of our study are summarized as follows: 1) Due to the retrospective character of the study, no study protocol had been followed and fluid management in the operating room had been performed according to the individual anesthesiologist being in charge. Also, there might have been a learning curve effect for the anesthesiologists, as the reported cases were the first ones in our institution. Therefore, values of the administered fluids in our study have a vast range (1000 – 8000 ml for crystalloids, 0 – 3500 ml for colloids). 2) The learning curve effect for the surgeons might have affected the rate of specific complications as well as the value of the estimated blood loss. 3) Postoperative complications were retrospectively collected from urologists. This might explain the extreme low PONV incidence compared to other studies [28]. In addition, complications were not documented using a predefined score such as described by Dindo et al. [29]. 4) The collection of data for our study focused exclusively on perioperative fluid management and its influence on outcome. We did not analyze the effect of preexisting disease in our patients and are therefore not able to determine, whether there is an interaction between preexisting disease, intraoperative fluid administration and outcome.

## Conclusions

The findings of our retrospective study underline the already known impact of intraoperative application of crystalloids and colloids on the rate of specific complications, this time demonstrated in patients undergoing RALP, representing a laparoscopic technique with a limited loss of fluids. We could show that crystalloids are associated with a higher incidence of leakage at the bladder-urethra anastomosis in older patients. Colloid administration to the same patients also showed a prolongation of the length of hospitalization with a potentially large impact on hospital economics. Further prospective and randomized trials have to be conducted to allow more detailed statements and to develop protocols for standardization of fluid replacement during RALP.

## Competing interests

The authors declare that they have no competing interests.

## Authors’ contributions

TP analyzed the data and wrote the manuscript, PD helped conceive the study and collect data, SG and SRH performed statistical analysis, DMS helped conduct the study and collect data, BBS conceived the study, analyzed the data and wrote the manuscript. Final manuscript was read and approved by all the authors.

## Pre-publication history

The pre-publication history for this paper can be accessed here:

http://www.biomedcentral.com/1471-2253/14/61/prepub
